# Quantification of gait changes in subjects with visual height intolerance when exposed to heights

**DOI:** 10.3389/fnhum.2014.00963

**Published:** 2014-12-04

**Authors:** Roman Schniepp, Günter Kugler, Max Wuehr, Maria Eckl, Doreen Huppert, Sabrina Huth, Cauchy Pradhan, Klaus Jahn, Thomas Brandt

**Affiliations:** ^1^Department of Neurology, University of MunichMunich, Germany; ^2^German Center for Vertigo and Balance Disorders (DSGZ), University of MunichMunich, Germany; ^3^Institute for Clinical Neurosciences, University of MunichMunich, Germany

**Keywords:** gait, visual height intolerance, dual task, visual input, gait variability

## Abstract

**Introduction:** Visual height intolerance (vHI) manifests as instability at heights with apprehension of losing balance or falling. We investigated contributions of visual feedback and attention on gait performance of subjects with vHI.

**Materials and Methods:** Sixteen subjects with vHI walked over a gait mat (GAITRite®) on a 15-m-high balcony and at ground-level. Subjects walked at different speeds (slow, preferred, fast), during changes of the visual input (gaze straight/up/down; eyes open/closed), and while doing a cognitive task. An rmANOVA with the factors “height situation” and “gait condition” was performed. Subjects were also asked to estimate the height of the balcony over ground level. The individual estimates were used for correlations with the gait parameters.

**Results:** Study participants walked slower at heights, with reduced cadence and stride length. The double support phases were increased (all *p* < 0.01), which correlated with the estimated height of the balcony (*R*^2^ = 0.453, *p* < 0.05). These changes were still present when walking with upward gaze or closure of the eyes. Under the conditions walking and looking down to the floor of the balcony, during dual-task and fast walking, there were no differences between the gait performance on the balcony and at ground-level.

**Discussion:** The found gait changes are features of a cautious gait control. Internal, cognitive models with anxiety play an important role for vHI; gait was similarly affected when the visual perception of the depth was prevented. Improvement by dual task at heights may be associated by a reduction of the anxiety level.

**Conclusion:** It is conceivable that mental distraction by dual task or increasing the walking speed might be useful recommendations to reduce the imbalance during locomotion in subjects susceptible to vHI.

## Introduction

The individual responses provoked by visual height stimulation comprise a broad spectrum ranging from the mildest response, a physiological postural height imbalance, to the severest, the specific phobia of acrophobia. Acrophobia can be described as a specific phobia of height situations with symptoms of a panic attack leading to avoidance behavior and psychological and psychosocial impairment (Kapfhammer et al., 2014). As a more intermediate condition that does not fulfill the criteria of a specific phobia, visual height intolerance (vHI) is a condition associated with the apprehension of losing balance and fear of falling when subjects are exposed to heights. Visual height intolerance is a frequent phenomenon affecting approximately 28% of the general population (Huppert et al., 2013; Brandt and Huppert, 2014). A major complaint of these subjects is a disturbance of stance and gait when they are exposed to heights and the fear to fall down into the depth. The visual height stimulus itself is thought to directly trigger these vHI-related impairments of stance and gait control. This assumption is based on earlier findings that postural imbalance at heights originates from a mismatch between the perception of self-motion relative to the surrounding environment when the distance between the nearest visible objects and the eyes reaches a critical threshold (Brandt et al., 1980; Salassa and Zapala, 2009). Accordingly, both, subjects unsusceptible to vHI (Bles et al., 1980) and subjects susceptible to vHI (Wuehr et al., 2014) presented an improved postural stabilization under height exposure when nearby stationary contrasts were present in their visual field. The distance to the surrounding at heights is dependent on the head position and the direction of the gaze in space. This suggests that the intensity of vHI during gait might be dependent on the direction of the gaze and the head position. Kugler et al. described different gaze-in-space strategies in heights for subjects with and without vHI. Compensation strategies might be represented in the findings of Kugler et al.; they reported subjects susceptible to vHI having a reduced visual exploration behavior and a frozen gaze in the straight ahead direction with intermittent fast eye movements toward the ground while walking on a high balcony (Kugler et al., 2014). In addition to an impaired visual exploration, an anxiety-driven motor program is thought to be triggered by the height stimulus that intensifies attention on body sway and muscle co-contraction, a process that may contribute to the impaired postural control (Brandt and Huppert, 2014; Wuehr et al., 2014).

The aim of the study was to investigate the role of altered visual feedback and attention on the gait performance of subjects with vHI when exposed to heights. Therefore, (i) the walking speed, (ii) the visual stimulus, and (iii) the capacity for attention were experimentally modified while the subjects walked on a high emergency escape balcony or at ground-level in the laboratory.

Visual modifications included changes of head/and gaze positions in the pitch plane and walking with eyes open or closed, thus causing experimental alterations in the perception of optic flow, verticality, and the overall awareness of height. In the second part of the experiment, dual-task paradigms were used to investigate the attentional demand of the walking task and to distract the subjects from the height situation.

The identification of visual and cognitive gait control principles for vHI may help develop coping strategies like those used to manage acrophobia. Such strategies may serve as important new components of the self-efficacy beliefs of subjects susceptible to vHI.

## Methods

### Patients

The cohort recruited for the measurement is identical with that of a previous study on balance control when standing still at heights (Wuehr et al., 2014). Sixteen subjects (7 females; mean age 46 ± 15 years; mean height: 1.74 ± 0.11 m; mean weight: 75.9 ± 18.3 kg) who had reported lifetime vHI participated in the study. A detailed questionnaire was used to enquire about vHI (Huppert et al., 2013). Subjects with acrophobia (to the extent of a specific phobia) or with past or current history of other psychiatric disorders (e.g., anxiety disorders, depression, or phobias) were excluded, as were participants with a known history of vestibular and balance deficits or other reported neurological or orthopedic disorders that affect gait. All selected participants had normal or corrected to-normal vision. The study protocol was approved by the local Ethics committee and has been performed in accordance to the ethical standards laid down in the 1964 Declaration of Helsinki and its later amendments. All participants gave their written informed consent prior to the experiments.

### Questionnaires

All subjects were asked to complete a custom-designed questionnaire. This included a subjective rating of the fear felt during height exposure (scale from 0 [no fear] to 10 [most severe form of fear]), the individual estimation of the height above ground level of the balcony (in meters) and a report of any accompanying somatic symptoms and compensatory behavior. Furthermore, the impact of vHI on daily activities and quality of life was evaluated.

### Gait assessment

Gait analysis was performed using a 6.7-m-long pressure-sensitive carpet (GAITRite®, CIR System, Havertown, USA) with a sampling rate of 120 Hz. The following gait conditions were examined in a fixed order: single task walking with preferred speed (STPS), single task walking with slow speed (STSS), single task walking with maximally fast speed (STMS), walking with extension of the head (45°, gaze upwards) with preferred speed (EHPS), walking with flexion of the head (45°, gaze downwards) with preferred speed (FHPS), dual task walking (serial 7 task) with preferred speed (DTS7), dual task walking (verbal fluency task) with preferred speed (DTVF), eyes closed walking with preferred speed (ECPS), and single task walking with preferred speed (STPS2). For the cognitive dual task paradigms, the subjects were instructed to focus on the cognitive task (cognitive task priorization).

Each walk was started 1.5 m in front of the mat and continued for 1.5 m beyond it in order to allow steady-state locomotion. Each task was tested twice. This gait assessment protocol was performed first on an emergency balcony (15 m/49.2 ft above ground) and afterwards in an indoor routine gait laboratory at ground level. The walking times for each condition ranged between 19 and 44 s on the balcony. In the laboratory, walking times ranged from 15 to 37 s.

### Data analysis

As the Wilcoxon and Mann-Whitney-U test revealed no significant side asymmetries for the different gait parameters and the different conditions, data of both limbs were pooled together in order to increase the number of steps and improve the quality of the CV values. Pooling the data yielded on average 18.3 ± 2.1 steps for STPS, 25.6 ± 4.2 steps for STSS, 14.5 ± 2.1 steps for STMS, 20.3 ± 3.2 steps for EHPS, 18.6 ± 1.7 steps for FHPS, 18.4 ± 2.4 steps for DTS7, 18.8 ± 2.0 steps for DTVF, 22.3 ± 3.8 steps for ECPS, 18.1 ± 2.2 steps for STPS2. Thus, the numbers of step events have undergone a critical number of 50 steps proposed by another group to assess gait variability with a high accuracy (Konig et al., 2014). However, multiplications of walks in order to reach this number of events would have meant an immense increase of experiment duration, which in turn would be prone to training effects.

CV values were calculated by using the formula:

$$\text{CV}[\%]=\frac{standard\;deviation\;of\;parameter}{mean\;of\;parameter}\times 100$$

To quantify the relative dual task cost, a Variation Rate (VR) on analogy to the Romberg quotient (Gagey and Weber, 1999) was calculated with the formula:

$$\text{VR}[\%]=\frac{parameter\;dual\;task–\;parameter\;single\;task}{\text{parameter single}}\times 100$$

The effects of each dependent variable were analyzed using a two-way repeated measurement analysis of variance (ANOVA) with the factors height stimulus (balcony vs. laboratory) and gait condition (STPS, STSS, STMS, EHPS, FHPS, DTS7, DTVF, ECPS, STPS2). The covariates age, gender, height, leg length were included into the model. If the ANOVA indicated a significant height stimulus x gait condition effect, a simple main effects test was performed to determine the degree to which one factor is differentially effective at each level of the second factor. *Post-hoc* analysis was carried out using a Bonferroni correction. Correlations between the subjective ratings of the fear of height, the estimates of the height of the balcony and the gait parameters were calculated using Pearson's and Kendall-Tau procedures. Matlab® and SPSS® were used for data analysis. In all tests, a *p*-value less than 0.05 was considered significant.

## Results

### Characteristics of the enrolled subjects

Subjectively rated anxiety while standing on the balcony averaged 4.6 ± 2.5 (min = 0; max = 10). The mean value of the subjective estimation (of each participant) of the height of the balcony was 16 ± 6 m (min = 6; max 40), respectively 52 ± 20 ft (min = 20; max = 131 ft). Among the accompanying somatic symptoms, instability of stance/gait was most frequently reported (64%), followed by inner agitation (57%), to-and-fro vertigo and trembling (both 43%), queasy-stomach feeling (36%), weakness in the knees (29%), fearfulness and sudden sweating (both 21%), palpitations and light headedness (14%). The most common compensatory behavior was thinking about a safe grip (57%), avoiding looking down (43%), staying close to the building walls (29%), and considering withdrawing from the experiment (21%). A total of 36% of the participants indicated that aside from typical triggering situations, vHI had no disturbing impact on their daily routine. On the other hand, 64% reported that in typical triggering situations vHI led to compensatory behavior with relevant consequences for their quality of life.

### Gait characteristics of subjects susceptible to vHI

None of the examined walking parameters showed any significant training effect for the duration of the experiments. The walking performance during height exposure was characterized by a reduction of walking speed, cadence, stride length, and swing phases (all *p* < 0.01), whereas stance phases, stride time, double support phases, and variability markers were increased (all *p* < 0.01) (for overview, Table 1, raw data in Tables 1, 2 of the supplemental files).

**Table 1:** **Gait changes of subjects susceptible to vHI**.

**Gait domain**	**Parameter**	**Mean value (STPS, balcony)**	**Mean value (STPS, laboratory)**	**Height (balcony/laboratory)**	**Condition**	**Height x Condition**
Pace/	Velocity [m/sec]	1.03 ± 0.08	1.26 ± 0.07	*F*_(1, 15)_ = 22.8, *p* = 0.0003	*F*_(1, 7)_ = 12.5, *p* = 0.0094	*F*_(7, 31)_ = 11.2, *p* = 0.0013
Rhythm	Cadence [m^−1^]	103 ± 5	117 ± 4	*F*_(1, 15)_ = 15.0, *p* = 0.0014	*F*_(1, 7)_ = 15.1, *p* = 0.0003	F_(7, 31)_ = 16.7, *p* = 0.0002
	Stride length [m]	1.15 ± 0.05	1.33 ± 0.05	*F*_(1, 15)_ = 12.8, *p* = 0.0028	*F*_(1, 7)_ = 25.6, *p* = 0.0000	*F*_(7, 31)_ = 31.9, *p* = 0.0000
Cycle	Stride time [s]	1.20 ± 0.06	1.02 ± 0.04	*F*_(1, 15)_ = 6.1, *p* = 0.0264	*F*_(1, 7)_ = 10.4, *p* = 0.0012	*F*_(7, 31)_ = 9.0, *p* = 0.0019
Support	Double support percentage [%]	31.2 ± 1.4	22.6 ± 1.0	*F*_(1, 15)_ = 43.2, *p* = 0.0000	*F*_(1, 7)_ = 24.5, *p* = 0.000	*F*_(7, 31)_ = 21.5, *p* = 0.0001
	Base of support [m]	0.11 ± 0.01	0.09 ± 0.01	*F*_(1, 15)_ = 25.8, *p* = 0.0001	*F*_(1, 7)_ = 6.8, *p* = 0.0051	*F*_(7, 31)_ = 6.7, *p* = 0.0050
Variability	CV of stride time [%]	3.1 ± 0.3	2.4 ± 0.2	*F*_(1, 15)_ = 11.1, *p* = 0.0046	*F*_(1, 7)_ = 6.2, *p* = 0.0073	*F*_(7, 31)_ = 3.7, *p* = 0.0377
	CV of stride length [%]	2.9 ± 0.4	2.5 ± 0.3	*F*_(1, 15)_ = 27.5, *p* = 0.0001	*F*_(1, 7)_ = 30.6, *p* = 0.0000	*F*_(7, 31)_ = 21.9, *p* = 0.0000
	CV of base of support [%]	18.6 ± 2.6	19.2 ± 2.0	*F*_(1, 15)_ = 0.4, *p* = 0.5550	*F*_(1, 7)_ = 2.6, *p* = 0.0888	*F*_(7, 31)_ = 2.8, *p* = 0.0755

*Table 1 depicts the results of the two-way ANOVA model with Bonferroni post-hoc analysis for height stimulus and gait conditions. The parameters were described as mean values and standard errors, grouped into gait domains on the basis of Lord et al. (2013). The results of the ANOVA model are presented for the factors “height” and “condition,” and for the interaction effect “height x conditions.” The main factors have significant effects on all examined gait parameters except of the mediolateral variability marker (CV of base of support), as does the interaction factor “height x condition.” Further results of the decomposition of the interaction factor are presented in Table 2*.

*STPS, single task walking with preferred speed; CV, coefficient of variation*.

Decomposition of the significant interaction effect revealed that height stimulus–related reductions of velocity, cadence, and stride length were only present in the walking conditions STPS, STSS, EHPS, ECPS (Figure **2**). Changes of the cycle/phase domain parameters were present in all walking conditions except STMS and FHPS (Table 2). EHPS and ECPS were the gait conditions with the most pronounced walking impairments (indicated by greater mean differences and higher *F*-values). These two conditions also showed significantly increased support and variability markers during height exposure (Table 2).

**Table 2:** **Decomposition of the effect of the current walking condition on the gait performance**.

		**Pace/Rhythm**	**Cycle/Phase**	**Support**	**Variability**
**GAIT CONDITIONS**					
Walking speed	Preferred	^↓^	^↑^	^↔^	^↔^
	Slow	^↓^	^↑^	^↔^	^↔^
	Maximally fast	^↔^	^↔^	^↔^	^↔^
Head maneuver	Extension	^↓^	^↑^	^↑^	^↑^
	Flexion	^↔^	^↔^	^↔^	^↔^
Dual tasks	Serial 7	^↔^	^↔^	^↔^	^↔^
	Verbal fluency	^↔^	^↔^	^↔^	^↔^
	Eyes closed	^↓^	^↑^	^↑^	^↑^
	Preferred 2	^↔^	^↑^	^↔^	^↔^

*Table 2 depicts the results of the decomposition for the interaction effect “height x condition.” Gait parameters were grouped into gait domains on the basis of Lord et al. (2013)*.

↓ *indicating significant reduction (balcony vs. laboratory)*.

↑ *indicating significant increase (balcony vs. laboratory)*.

↔ *indicating no significant change (balcony vs. laboratory)*.

*Pace domain: gait velocity, stride length*.

*Rhythm domain: cadence, stride time*.

*Phase domain: single and double support phases, swing phases, stance phases*.

*Variability domain: coefficient of variation of stride length and of stride time*.

*Support domain: base of support, coefficient of variation of base of support*.

For the dual task conditions, decomposition of the interaction effect revealed no significant changes between the two height situations (Table 2). Calculation of the dual task cost showed that the percentage of gait parameter changes (DTc to preferred walking) was significantly smaller in the height situation compared to the laboratory situation (e.g., DTS7; velocity −8.2 ± 2.3% in height situation compared to −18.3 ± 3.3% in the laboratory, *p* < 0.05) (Figure **3**).

**Figure 1:**
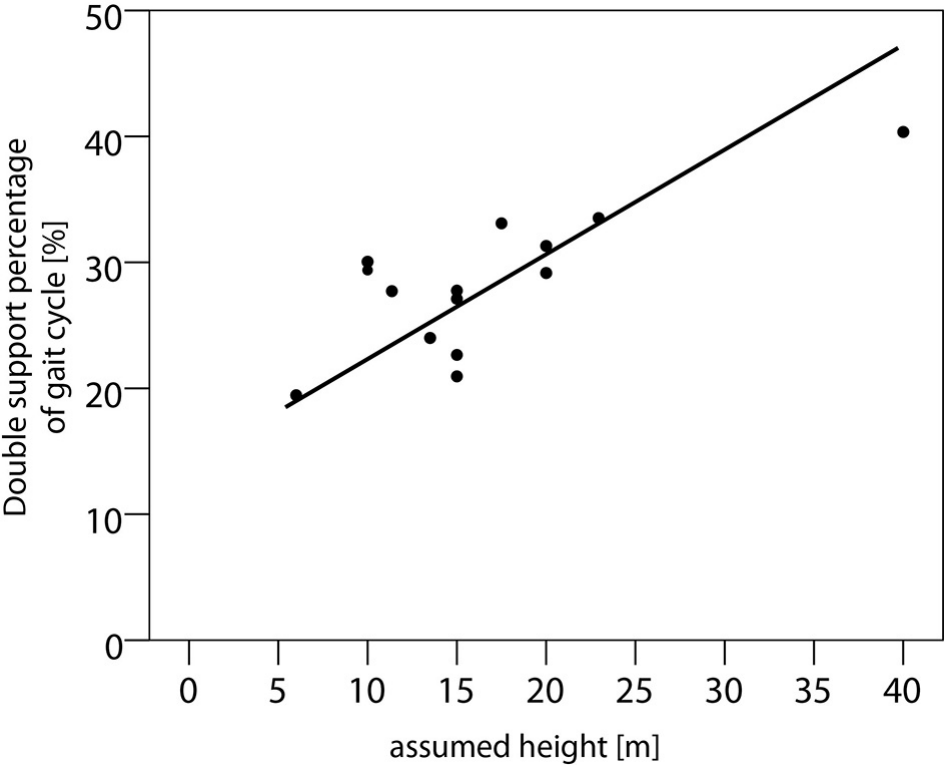
**Relationship of the assumed height of the balcony above ground level and double support phases in vHI (preferred walking)**. Scatter plot of the assumed heights (in m) and the percentage of double support phases with respect to the gait cycle of the vHI subjects. Pearson's correlation revealed a *R*^2^ = 0.453 (*p* < 0.05) for the gait condition of preferred walking (displayed in this graph).

Pearson's correlation found a significant correlation between the assumed height (in meters) of the balcony and the double support percentage for the walking conditions STPS, EHPS, and ECPS (Figure 1; preferred walking) and the walking velocity for the walking conditions EHPS and ECPS (*R*^2^ values are summarized in Table 3).

**Figure 2:**
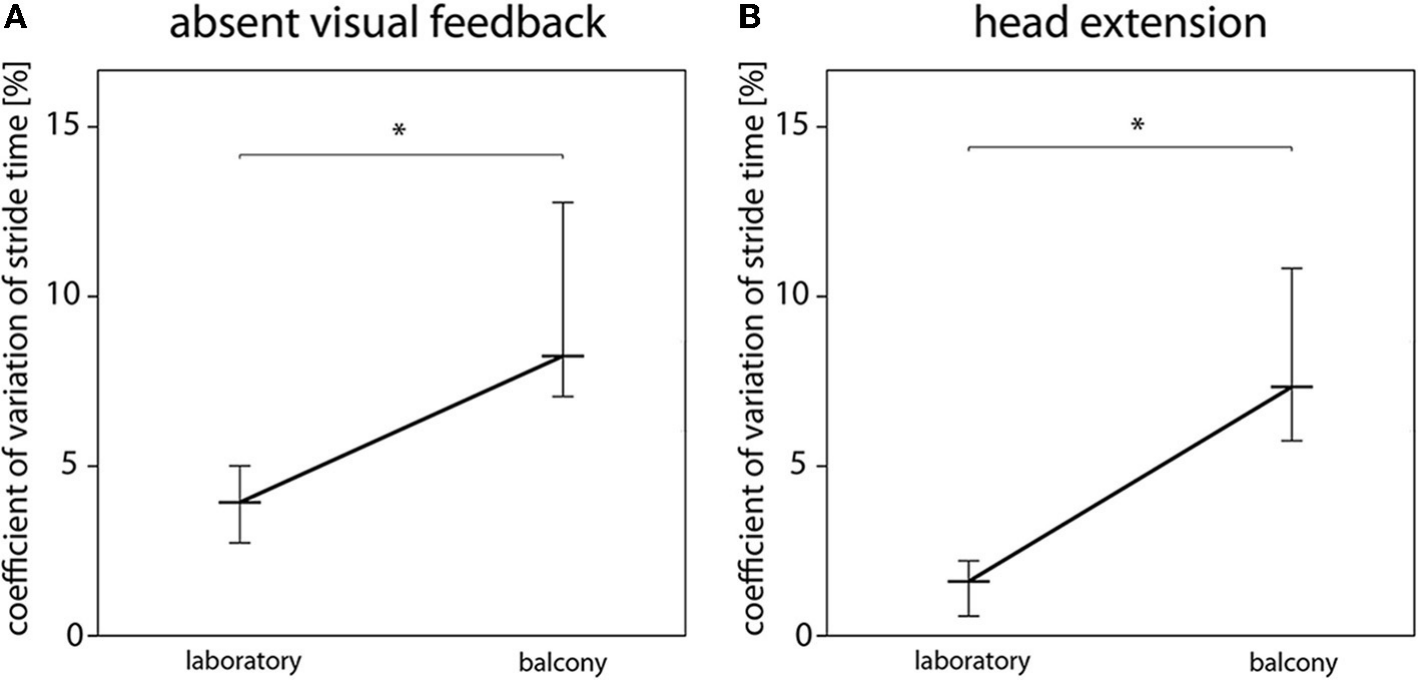
**Influence of absent visual feedback and head position on temporal gait variability**. The magnitude of temporal gait variability during walking with eyes closed (absent visual feedback) **(A)** and walking with extension of the head **(B)** depends on the height stimulus. The coefficient of variation of stride time is significantly increased in the balcony situation. ^*^indicates *p* < 0.05. Black, bold lines indicate the medians of the coefficient of variation of stride time [%] for all vHI subjects (black thin lines represent the 0.95 confidence interval).

**Figure 3:**
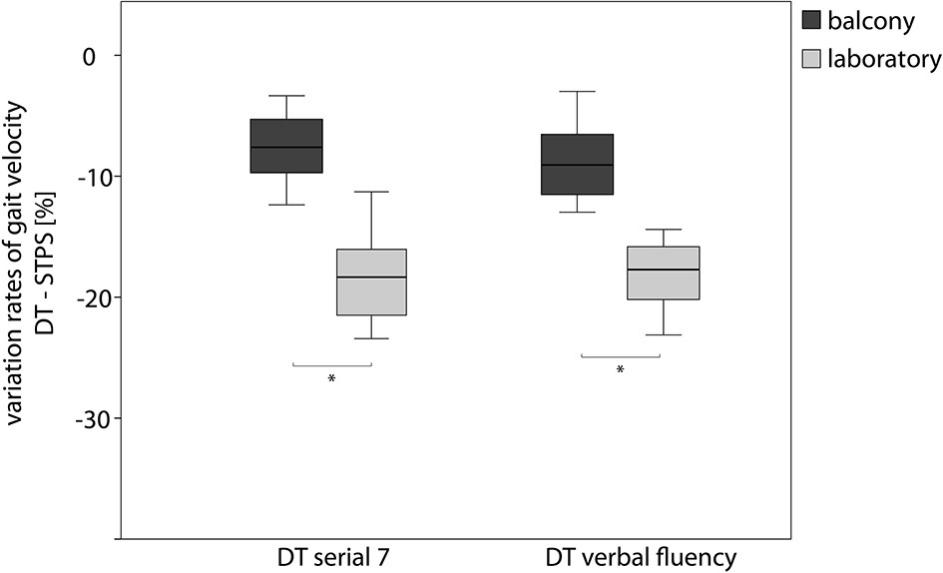
**Effect of a cognitive loading on the gait velocity during walking on the balcony and in the laboratory**. Boxplot graph of the individual Variation Rates (percentage of decrease) for gait speed during walking and performing a Serial 7 Dual Task and walking and performing a Verbal Fluency Dual Task. Dark gray boxplots represent the Dual Task costs on the balcony, light gray in the basement laboratory. DT, Dual Task; STPS, Single Task walking with preferred speed. ^*^indicates *p* < 0.05.

**Table 3:** **Correlation of the assumed height of the balcony above ground level and gait parameters**.

		**Velocity**	**Stride length**	**Double support percentage**	**CV of stride time**	**Base of support**
**GAIT CONDITIONS**						
Walking speed	Preferred	n.s.	n.s.	0.453^*^	n.s.	n.s.
	Slow	n.s.	n.s.	n.s.	n.s.	n.s.
	Maximally fast	n.s.	n.s.	n.s.	n.s.	n.s.
Head maneuver	Extension	0.71^**^	n.s.	0.65^**^	n.s.	n.s.
	Flexion	n.s.	n.s.	n.s.	n.s.	n.s.
Dual tasks	Serial 7	n.s.	n.s.	n.s.	n.s.	n.s.
	Verbal fluency	n.s.	n.s.	n.s.	n.s.	n.s.
	Eyes closed	0.83^**^	n.s.	0.61^**^	n.s.	n.s.
	Preferred 2	n.s.	n.s.	n.s.	n.s.	n.s.

*Results from the Pearson's coefficient correlation analysis for several gait parameters and the assumed height of the balcony in meters (estimated by each individual standing on the balcony). Double support and gait velocity showed significant correlations with the assumed height of the balcony. These correlations were maximal during gait conditions with altered visual field perception (head extension and gaze upwards, eyes closed)*.

* p < 0.05;

** *p < 0.01*.

## Discussion

Our main findings are as follows:

- The gait performance of subjects susceptible to vHI exposed to heights is characterized by a reduction of walking speed, stride length, and increased double support phases. Such changes are typical features of cautious gait control. This cautious gait strategy is overridden when vHI subjects walk at a maximally fast speed.
- Even when the visual stimulus of depth is absent (eye closure, head extension), the gait impairments do not improve. Instead, they are associated with increased gait variability, a marker for dynamic instability.
- Mental distraction during locomotion at heights normalizes the gait performance of subjects with vHI, which might be a helpful coping strategy and might improve the self-efficacy of the subjects.

### Gait impairments while walking with head upright

When exposed to heights, subjects susceptible to vHI walked slower, with a reduced cadence, reduced stride length and increased double support phases. These changes fit well to the concept of a “cautious gait” control, which is an established term in the field of geriatric gait research (Giladi et al., 2005; Herman et al., 2005). Cautious gait is a higher-level gait disorder in which the walking strategy is impaired by a fear of falling (Aizen, 2001). Pace reduction may be a safety strategy of the walker to prevent falls, since it co-influences the variables of rhythm and cycle domains in terms of a prolongation of double support phases. The fear of falling in patients with phobic postural vertigo correlated with the reduction of walking speed, thus indicating that anxiety and the speed of performance are directly related in this condition (Schniepp et al., 2014). This direct relationship was not present in the current study, as the subjective overall anxiety of vHI did not correlate with gait measures. This finding is comparable to results of an earlier study (Kugler et al., 2014). There was, however, a positive correlation for the duration of double-support phases and for the walking speed with the individual estimates of the height of the balcony above ground. Interestingly, there is evidence that height-fearful subjects optically overestimate height situations (Stefanucci and Proffitt, 2009). Thus, it remains unclear whether the individually perceived intensity of the height situations directly influences gait control or whether this relationship is a consequence of a biased estimation.

The increase of co-contractions of antigravity muscles, which has been found in subjects susceptible to vHI standing still on a high balcony (Wuehr et al., 2014), may additionally play a role for the speed reduction of gait. During gait, the co-contraction of agonist and antagonist muscles occur when the instability of a joint movement exceeds a critical threshold; elevated coactivation of antagonist muscles has thus been described as a stiffness and safety strategy (Hortobágyi et al., 2009) and there is first evidence for the relationship between the fear of falling of elderly walkers and the occurrence of co-contractions of leg muscles (Nagai et al., 2012).

Gait variability parameters were normal in subjects susceptible to vHI when they walked with the head in a natural position or while performing a cognitive dual task. Gait variability, i.e., the intrinsic fluctuations within the walking patterns, contains relevant information about the dynamic instability of a walking pattern (Hausdorff et al., 1997a; Hausdorff, 2005; Schniepp et al., 2012; Wuehr et al., 2013). Variability measures are linked to the fall risk, which has been shown for elderly subjects (Hausdorff et al., 1997a), for patients with basal ganglia pathologies (Hausdorff et al., 1998; Hausdorff, 2009), and for patients with cognitive impairments (Sheridan et al., 2003). According to patients with phobic postural vertigo, subjects susceptible to vHI did not show increased magnitudes of gait variability which indicates a preserved capacity to avoid falls (Schniepp et al., 2014), even if the fear of falling is pronounced in the subjects.

When the subjects susceptible to vHI were asked to walk fast, no significant differences between the balcony and the laboratory situation were present, which indicates that the cautious gait control can be overcome by walking fast. This is in accordance to parallel findings in patients with phobic postural vertigo (Schniepp et al., 2014) and might be a result of a higher automated walking control during fast walking when visual-vestibular feedback is less important.

Intercondition effects for walking at the beginning and at the end of the experiment were insignificant as were the interaction effect for “height situation × gait condition,” indicating that there was no significant habituation process in the gait performance for both experimental situations.

The above-described alterations make up the basic pattern of vHI gait at heights. In the following, we are focusing on the variations of this gait pattern under different gait conditions.

### Eye closure, direction of gaze, and the head position influence vHI gait

**Figure 4:**
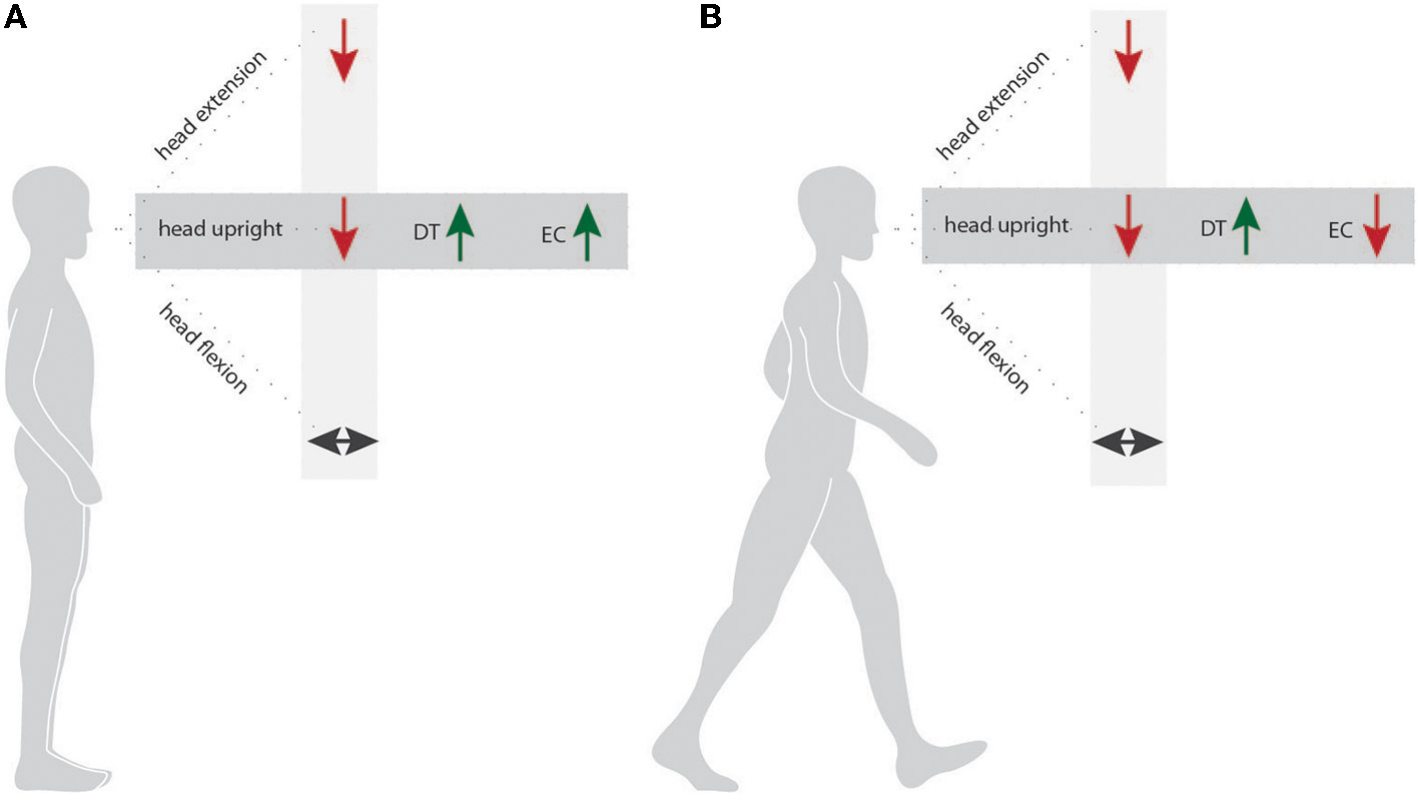
**Modulation of stance and gait behavior induced by different postural tasks**. Figure 4 presents a schema summarizing the effects of the factors “height situation” and “postural condition” on the stance performance **(A)** and gait performance **(B)** of subjects susceptible to vHI. Light gray sector—condition effect for “height condition” (balcony compared to laboratory). Dark gray sector—condition effect for “postural condition” (Dual Task (DT) or Eye Closure (EC) compared to Single Task). 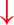 indicates significant deterioration of stance/gait performance. 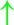 indicates an improved stance/gait performance. ↔ indicates no significant differences.

Visual feedback appears to have two opposite effects for subjects susceptible to vHI when walking at heights: while visual feedback is essential for maintaining dynamic stability during walking (Wuehr et al., 2013), the accompanying perception of visual depth stimuli has been shown to impair postural stability and trigger anxiety in vHI subjects (Bles et al., 1980; Chou et al., 2009; Wuehr et al., 2014). In order to test particular parameters of visual perception in heights, we investigated the gait performance of vHI when they were walking with different gaze and head positions or with eye closure. Extension of the head and gaze toward the underpart of the emergency balcony one level above the experimental site were used to investigate the walking capability with visual inputs of optical flow, but with elimination of visual information about the depth. Walking with eyes closed eliminated the visual exposure to height while retaining full knowledge about the potential risk of falling. Remarkably, none of these conditions attenuated the gait impairments of vHI. Even when the visual perception of the depth of the balcony removed (head extension, eye closure), the cautious walking strategy of vHI was still present (for overview, Figure 4). Here the interaction effect (height situation × gait conditions) was significant and walking speed, cadence, and stride length decreased more at heights than at ground level in the laboratory. Moreover, these conditions were the only ones associated with increased gait variability parameters which in turns points toward an increased risk for falling and disturbed dynamic stability under such circumstances (Hausdorff et al., 1997a). Thus, the gait alterations of vHI are not exclusively provoked by insufficient self-motion perception or disparity of optical flow. Instead, we can assume a higher-level pathomechanism. The visual depth stimuli might reflexively activate an “anxiety program” at the first encounter of the height situation, which continues to persist, even when the visual perception of the depth is withdrawn. Tersteeg and colleagues concluded that the mechanisms disturbing locomotion, balance, and autonomic response during height exposure occur at a high task level, in which cognition of prior experience is integrated with sensory inputs (Tersteeg et al., 2012). Our results are in line with those of a previous investigation of gaze behavior during locomotion (Kugler et al., 2014), which found that gaze straight ahead and fast eye movements toward the ground predominated in these subjects.

In contrast to head extension and eyes closure, walking with a flexed head and gaze toward the ground preserves visual perception of the surface just in front of the walker. During this condition, we observed only minimal walking impairments in vHI subjects, indicating that such gaze maneuvers might have a stabilizing effect while walking at heights.

### Dual task paradigms improve the gait performance

A current concept of postural control is that it shares attentional resources with cognition (Kerr et al., 1985). Dual task paradigms, employing walking and cognitive tasks simultaneously, have been used to investigate motor and cognitive interference during gait (Woollacott and Shumway-Cook, 2002). The main theoretical models accounting for dual task interference are (i) the capacity- or resource-sharing model and (ii) the cross-talk model (Pashler, 1994) for a detailed review). Gait deterioration under cognitive dual tasks has been found in elderly subjects prone to falls (Beauchet et al., 2005; Kressig et al., 2008), patients with cognitive impairments (Allali et al., 2007; Beauchet et al., 2011), and patients with basal ganglia pathologies (Hausdorff et al., 2003). The capacity-sharing model interprets these findings to indicate a depletion of available attentional resources. For subjects susceptible to vHI, dual task conditions were characterized by the absence of significant gait differences between the balcony and laboratory situations. This paradox, namely that the height stimulus has no effect on gait during a dual-task paradigm, supports the cross-talk model or the concept of interference between the motor and cognitive tasks in vHI. This interference could occur directly and indirectly through (i) a reduction of attentional efforts spent for the gait control, or (ii) an affection of the emotional/ mental processing of the height situation. The latter has been described as a possible mental distraction from the anxiety-eliciting stimuli. Mental distraction has been found as a self-efficient coping strategy in acrophobia (Marshall et al., 1992), and is applied in the initial phases of behavioral therapy approaches for this condition, e.g., during self-directed exposure or desensitization processes (Marshall, 1981). Such methods might be promising and effective control strategies, particularly for conditions with mild anxiety-driven symptoms (like vHI) that do not fulfill the criteria for a specific phobia.

### Differential aspects for stance and gait in vHI

The cohort investigated in this study was the same as that examined in a former study by our group (Wuehr et al., 2014). In this study, static posturographic recordings were combined with surface electromyography measurements of leg, arm and neck muscles. The protocol likewise used an experimental procedure to investigate stance behavior during changes of the head/ and gaze direction, while standing with eyes closed, or standing under single-task and dual-task conditions. Taken together, this previous study and the present findings indicate that the performance of a cognitive dual task improved the stability of the individuals susceptible to vHI during stance and gait when exposed to heights. This suggests that a higher attentional, anxiety-driven control can be distracted by an easy cognitive task during height exposition. The consequences of changes of the visual feedback appear to be different for stance and gait (for overview Figure 4). It was beneficial for stance and gait to fixate near targets in the periphery of the visual field or at ground level (by flexion of the head and gaze downwards). However, most meaningful were the differences between stance and gait at heights when the subjects closed their eyes. Stance improved during eye closure, indicating that the visual input directly triggers postural disequilibrium, for example by disturbing the fixation of targets or by perceiving the depth. In the walking task, however, visual perception of the height situation was not essential. The perception of the depth under the balcony (prior to the walking experiment) initiated a higher cognitive processing sequence that affected the gait of the subjects. Thus, the perception of the heading target and of the physiological optic flow information seems to be beneficial for stabilizing gait.

### Behavioral suggestions for subjects susceptible to vHI

Walking speed, gaze behavior, head position, and mental distraction influence the postural performance of subjects susceptible to vHI. In general, walking at an accelerated speed and fixating near targets straight ahead or at ground level seem to be beneficial, whereas extending the head and gazing upwards appear to worsen stance and gait control of the subjects. Closing the eyes (when securely supported) is only beneficial while standing at heights.

The most useful strategy for subjects susceptible to vHI is to mentally distract themselves from the height stimulus, e.g., by performing a second, easy dual task, either of verbal fluency or of executive function.

## Conclusion

This study provides first evidence for the underlying principles of impaired gait control of vHI exposed to heights. An allocentric, anxiety-related cognitive processing of the height situation initiates cautious gait control, which cannot be stopped by removing the visual perception of the height scene. Mental distraction by means of easy cognitive dual tasks can efficiently improve the walking capability of subjects susceptible to vHI. Thus, such strategies can be recommended to increase the self-assurance of these subjects.

## Funding

The work was supported by the German research foundation (Deutsche Forschungsgemeinschaft, DFG JA 1087/1-1), the German Hertie Foundation and the German Federal Ministry for Education and Science (BMBF, Nr. 01EO0901).

### Conflict of interest statement

The authors declare that the research was conducted in the absence of any commercial or financial relationships that could be construed as a potential conflict of interest.
