# Supplementary material for: Quantification of gait changes in subjects with visual height intolerance when exposed to heights
Source: Front Hum Neurosci. 2014 Dec 4;8:963. doi: 10.3389/fnhum.2014.00963 (PMC4255593; doi:10.3389/fnhum.2014.00963)
Supplement: Supplementary file 1 [file Table1.DOCX]

Table e-1 (supplemental file)

| Gait domain | Parameter | STPS | STSS | STMS | EHPS | FHPS | DTS7 | DTVF | ECPS | STPS 2 |
| --- | --- | --- | --- | --- | --- | --- | --- | --- | --- | --- |
| Pace/Rhythm | velocity [m/sec] | 1.03 ± 0.08 | 0.43 ± 0.05 | 1.86 ± 0.10 | 0.91 ± 0.07 | 1.05 ± 0.07 | 0.88 ± 0.09 | 0.89 ± 0.08 | 0.62 ± 0.06 | 1.01 ± 0.07 |
|  | cadence [m^-1^] | 103 ± 5 | 63 ± 5 | 148 ± 6 | 104 ± 5 | 105 ± 6 | 91 ± 7 | 92 ± 6 | 98 ± 5 | 103 ± 4 |
|  | stride length [m] | 1.15 ± 0.05 | 0.82 ± 0.03 | 1.48 ± 0.06 | 1.00 ± 0.06 | 1.14 ± 0.04 | 1.21 ± 0.09 | 1.11 ± 0.05 | 0.75 ± 0.05 | 1.15 ± 0.05 |
| Cycle | stride time [s] | 1.20 ± 0.06 | 2.08 ± 0.2 | 0.82 ± 0.03 | 1.17 ± 0.05 | 1.10 ± 0.06 | 1.91 ± 0.06 | 1.41 ± 0.12 | 1.26 ± 0.07 | 1.26 ± 0.05 |
| Support | double support percentage [%] | 31.2 ± 1.4 | 49.4 ± 7.0 | 17.8 ± 1.4 | 31.2 ± 2.1 | 30.1 ± 1.6 | 28.9 ± 1.7 | 29.9 ± 1.3 | 38.6 ± 2.7 | 33.2 ± 1.2 |
|  | base of support [m] | 0.11 ± 0.01 | 0.11 ± 0.01 | 0.11 ± 0.01 | 0.11 ± 0.01 | 0.11 ± 0.01 | 0.11 ± 0.01 | 0.11 ± 0.01 | 0.13 ± 0.01 | 0.11 ± 0.01 |
| Variability | CV of stride time [%] | 3.1 ± 0.3 | 6.9 ± 2.7 | 3.8 ± 0.5 | 9.3 ± 0.9 | 2.9 ± 0.4 | 4.8 ± 0.9 | 4.4 ± 0.6 | 8.7 ± 0.9 | 3.8 ± 0.4 |
|  | CV of stride length [%] | 2.9 ± 0.4 | 5.6 ± 2.7 | 2.4 ± 0.4 | 9.9 ± 1.2 | 3.5 ± 0.5 | 4.8 ± 1.3 | 4.0 ± 0.4 | 14.4 ± 0.8 | 3.3 ± 0.6 |
|  | CV of base of support [%] | 18.6 ± 2.6 | 17.9 ± 3.5 | 16.6 ± 1.6 | 20.6 ± 2.6 | 19.4 ± 2.2 | 18.5 ± 3.5 | 12.6 ± 2.6 | 28.9 ± 5.2 | 17.8 ± 2.5 |

Legend: Raw data of the gait performance of subjects susceptible to vHI when exposed to heights (15m high balcony).

The gait parameters were grouped into gait domains (according to Lord et al. 2013). Mean values and standard errors are presented.

Abbreviations: vHI - visual height intolerance, CV - coefficient of variation, STPS - single task walking with preferred speed, STSS - single task walking with slow speed, STMS - single task walking with maximally fast speed, EHPS - walking with extension of the head (45°, gaze upwards) with preferred speed, FHPS - walking with flexion of the head (45°, gaze downwards) with preferred speed, DTS7 - dual task walking (serial 7 task) with preferred speed, DTVF - dual task walking (verbal fluency task) with preferred speed, ECPS - eyes closed walking with preferred speed, STPS2 - single task walking with preferred speed at the end of the experiment.
